# Supplementary material for: Multi-omics analyses reveal that the gut microbiome and its metabolites promote milk fat synthesis in Zhongdian yak cows
Source: PeerJ. 2022 Dec 2;10:e14444. doi: 10.7717/peerj.14444 (PMC9744170; doi:10.7717/peerj.14444)
Supplement: Supplemental Information 13 [file peerj-10-14444-s013.zip › Web_Report/Metabolites_annotation/KEGG/All_kegg_map/ko05140.html]

ko05140
